# *MMP-2* gene polymorphisms are associated with type A aortic dissection and aortic diameters in patients

**DOI:** 10.1097/MD.0000000000005175

**Published:** 2016-10-21

**Authors:** Ou Liu, Wuxiang Xie, Yanwen Qin, Lixin Jia, Jing Zhang, Yi Xin, Xinliang Guan, Haiyang Li, Ming Gong, Yuyong Liu, Xiaolong Wang, Jianrong Li, Feng Lan, Hongjia Zhang

**Affiliations:** a Department of Cardiovascular Surgery, Beijing Lab for Cardiovascular Precision Medicine, Beijing Anzhen Hospital, Capital Medical University, Beijing, China; b Department of Epidemiology and Biostatistics, Imperial College London, London, UK; c Beijing Institute of Heart Lung and Blood Vessel Diseases, Beijing, China.

**Keywords:** aortic diameter, *MMP-2* gene, single nucleotide polymorphism, type A aortic dissection

## Abstract

Matrix metalloproteinases-2 (MMP-2) plays an important role in the pathogenesis of type A aortic dissection (AD). The aim of this study was to evaluate the association of 3 single nucleotide polymorphisms (SNPs) in the *MMP-2* gene with type A AD risk and aortic diameters in patients. We performed a case–control study with 172 unrelated type A AD patients and 439 controls. Three SNPs rs11644561, rs11643630, and rs243865 were genotyped through the MassARRAY platform. Allelic associations of SNPs and SNP haplotypes with type A AD and aortic diameters in patients were evaluated. The frequency of the G allele of the rs11643630 polymorphism was significantly lower in type A AD patients than in control subjects (odds ratio 0.705, 95% confidence interval 0.545–0.912, *P* = 0.008). The association remained significant after adjusting for clinical covariates (*P* = 0.008). Carriers of the GG genotype of the rs11643630 polymorphism had significantly smaller aortic diameters than those with GT genotype or TT genotype (*P* = 0.02). Further haplotype analysis identified 1 protective haplotype (GC; *P* = 0.008) for development of type A AD. Again, a significant correlation was observed between haplotype GC and AD size (*P* = 0.020). Our results suggest that *MMP-2* gene polymorphisms contribute to type A AD susceptibility. In addition, *MMP-2* gene SNPs are associated with AD size, which could be used as a target for the development of new drug therapy.

## 1 Introduction

Aortic dissection (AD) with an incidence of about 3 to 5 cases per 100,000 people per year remains a life-threatening event with a high mortality and significant long-term morbidity.^[1–3]^ Stanford type A AD originates primarily in the ascending aorta just above the aortic valve.^[4]^ In spite of the substantial advances made in diagnostic imaging methods and surgical techniques for treatment, the mortality of type A AD still averages 25%.^[5]^ Further difficulties in prevention and therapeutic management arise from the fact that the responsible molecular and genetic determinants of type A AD remain largely unidentified.

It is believed that genetic susceptibility is an important risk factor for type A AD.^[6]^ Up to 20% of type A AD patients have a first-degree relative with aortic disease.^[7]^ Many syndromes, such as Marfan syndrome and Loeys–Dietz syndrome, predispose individuals to familial forms of AD.^[8,9]^ However, the cause of nonsyndromic forms of type A AD remains unclear.

Gene expression studies have shown that, in type A AD, the expression of genes regulating extracellular matrix (ECM) remodeling are altered.^[10,11]^ It results in the important histopathological characteristics of type A AD: degenerative changes in the aortic wall.^[12]^ Genetic variations affecting gene expression which regulates ECM metabolism may increase the risk of type A AD.

Matrix metalloproteinases-2 (MMP-2) is of particular interest in AD because it is a product of mesenchymal cells including the smooth muscle cells of the aortic media.^[13,14]^ These cells are responsible for synthesis and maintenance of the complex macromolecular structure of the aorta.^[15]^ It has been demonstrated that MMP-2 plays a crucial role in pathogenesis of type A AD and some other tissue remodeling-related disease, for example, tumor.^[13,16]^ Recently, Beeghly-Fadiel et al^[17]^ identified that 3 single nucleotide polymorphisms (SNPs) in *MMP-2* gene were associated with breast cancer development. SNPs are the most common genetic variants in the human genome, which could explain differences in genetic susceptibility to complex disease.^[18–20]^ Type A AD is a complex trait which is assumed to be caused by genetic factors.^[21]^ In addition, it remains unclear whether these 3 SNPs that are associated with tumor could influence the formation of type A AD.

Prompted by these considerations, we carried out a case–control association study in a Chinese Han population to test the association of these 3 SNPs in *MMP-2* gene with type A AD. In an attempt to further explore the potential causal role of *MMP-2* gene on AD, we also investigated whether aortic diameters in type A AD patients could be influenced by the SNPs of the *MMP-2* gene.

The reason why we chose these 3 SNPs was that they were identified to be associated with breast cancer development in a previous study.^[17]^ Although breast cancer and type A AD were different diseases, they did have some similar pathological processes.^[14,22]^ They were both tissue remodeling–related diseases. In addition, degradation of ECM proteins by MMP-2 was a key mechanism in the initiation and progression of both cancer and AD.^[14,22]^ MMP-2 played a specific role in the development of both diseases. Based on these evidence, we hypothesized that these 3 SNPs of *MMP-2* gene identified to involve in pathogenesis of cancer should also contribute to the risk of type A AD.

## 2 Methods

### 2.1 Study subjects

We enrolled 172 unrelated cases that were randomly selected from patients admitted to Beijing Anzhen Hospital with a diagnosis of type A AD. The diagnostic criteria for type A thoracic AD (TAD) are as described previously.^[23]^ Type A AD is diagnosed using different imaging modalities such as computed tomography, echocardiography, magnetic resonance imaging, or angiography (Table 1). Patients with the bicuspid aortic valve or any other known aortic diseases such as Marfan syndrome and aortic coarctation were excluded from the study.

**Table 1:**
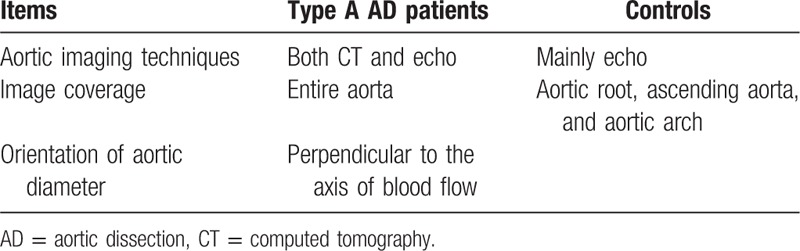
Aortic imaging techniques used for diagnostic evaluation.

Control subjects were recruited from the same hospital and consisted of patients who were admitted for reasons other than aortic disease, mainly primary hypertension disease. Control patients were enrolled only if angiography revealed no evidence of aortic diseases. Study participants were interviewed in person by trained medical professionals using a structured questionnaire. The definitions of risk factors such as hypertension, dyslipidemia, diabetes, coronary artery disease (CAD), tobacco, and alcohol use were as previously described.^[6]^ All subjects in this study were of the same ethnicity (Chinese Han).

Maximum axial aortic diameters in the ascending aorta were assessed by computed tomography or echocardiography at the time of presentation in AD patients (Table 1). Measurements were made at Beijing Anzhen Hospital and verified by an experienced investigator. This study was approved by the Institutional Review Board at Beijing Anzhen Hospital, and all subjects provided informed consent.

### 2.2 Genotyping

Ethylenediaminetetraacetic acid anticoagulated venous whole blood samples were collected from each TAD patient and control. Genomic deoxyribonucleic acid (DNA) was extracted using Qiagen DNA Purification kits (Qiagen, Hilden, Germany) according to manufacturers’ instructions. Genotyping of the SNPs was carried out using the Sequenom MassARRAY system (Sequenom, San Diego, California USA) that utilizes a homogenous MassExtend (hME—single base extension) reaction termed iPLEX GOLD. Genotyping quality was assessed by examination of duplicate concordance and call rates for each SNP and a test for compliance with Hardy–Weinberg equilibrium in controls.

### 2.3 Statistical analysis

All analyses were performed using the PLINK 1.07 software. Group differences in demographic and baseline clinical data were compared using chi-square tests in case of qualitative data and with 2-sample t tests for independent samples in case of quantitative data. Comparison of the distributions of the alleles was performed using the chi-square tests. Genetic association was also assessed under different genetic models with chi-square tests or Fisher exact tests. Where more than 5 counts for a given genotype were observed, a chi-square test was used; otherwise, Fisher exact test was used. Cochran–Armitage tests for trends were also implemented. Associations with type A AD risk were evaluated by computing odds ratios (ORs) and corresponding 95% confidence intervals (95% CIs). Bonferroni corrections for multiple comparisons were performed, and we used logistic regression analysis to test the independence of association evidence between SNPs and type A AD. Covariates considered included age, sex, smoking habit, diabetes, hypertension, and dyslipidemia. Aortic diameters in type A AD patients were tested for association using Wald test. Haplotypes were estimated using the Haploview 4.2 software (Harvard, Cambridge, MA USA). To correct for multiple testing in the haplotype analysis, 15,000 permutations were performed.

## 3 Results

### 3.1 Population demographics

Blood samples were obtained from 172 type A AD patients. Overall mean age was 50.0 years (±11.3). A total of 439 blood samples were obtained from people with no demonstrable AD on computed tomography scan. Mean age was 51.3 years (±14.4). We found a statistically significant gender difference in the occurrence of type A AD that was similar to previous reports.^[24]^ There were 121 (70.3%) males in the cases, compared to 222 (50.6%) in the controls. There were no significant differences between cases and controls in their risk factors such as alcohol intake and smoking habit as detailed in Table 2.

**Table 2:**
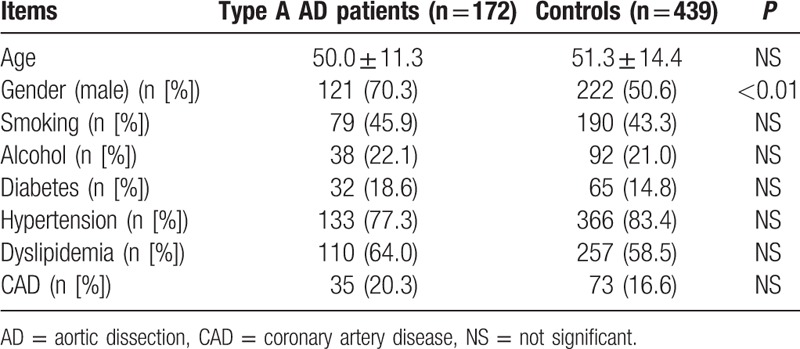
Demographic and clinical characteristics of both type A AD cases and controls in a Chinese Han population.

### 3.2 Association between the 3 SNPs in *MMP-2* gene and type A AD

As shown in Table 3, allele frequencies and distributions of 3 SNPs in type A AD patients and controls were reported. The frequency of the G allele of the rs11643630 polymorphism was significantly lower in type A AD patients than in control subjects (OR 0.705, 95%CI 0.545–0.912, *P* = 0.008). A statistically significant difference in genotype frequency distribution was found in AD patients as compared with controls according to the additive (*P* = 0.009) or dominant model (*P* = 0.002). Based on Cochran–Armitage tests, genotype distribution for rs11643630 also resulted in statistical differences between patients and controls (Table 3). The frequencies of the GG and GT genotypes were significantly lower in type A AD patients than in controls (*P* = 0.002). Furthermore, we performed a Bonferroni test to calculate the corrected *P* value. Again, the corrected *P* value was significant. However, no evidence for an association between the other 2 tested SNPs and the type A AD phenotype was detected either at the allele or at the genotype level.

**Table 3:**
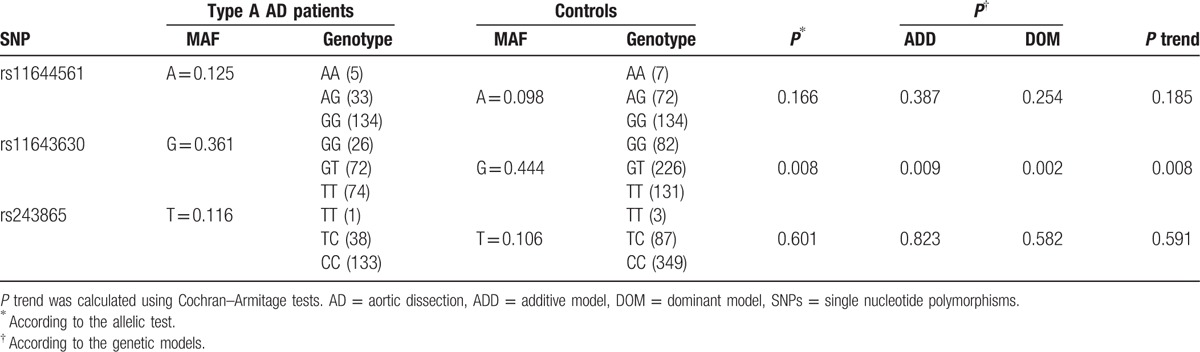
Allele frequency and genotype distribution of 3 investigated SNPs in type A AD patients and control subjects.

In the multiple logistic regression analysis with type A AD as dependent variable and age, gender, diabetes, hypertension, dyslipidemia, CAD, smoking, and alcohol habit as independent variable, the SNP rs11643630 in *MMP-2* gene resulted in an independent genetic susceptibility factor for type A AD (OR 0.698, 95% CI 0.536–0.909, *P* = 0.008).

Furthermore, we have conducted a more comprehensive study with the *MMP-2* gene SNPs discovered in our previous report.^[25]^ We found no significant association between these SNPs and the type A AD phenotype. In further multiple logistic regression analysis with age, gender, hypertension, diabetes, dyslipidemia, and smoking as the independent variables and type A AD as the dependent variable, no significant effect could be observed.

### 3.3 Aortic dissection size and MMP-2 genotype

Aortic diameters were measured in 172 patients with type A AD and in 439 controls. Aortic diameters of type A AD patients were significantly higher than those of controls (mean ± SD 52.20 ± 8.36 vs 32.81 ± 4.63 mm, *P* < 0.001). Aortic diameters were also analyzed in type A AD patients in relation to MMP-2 SNPs because MMP-2 was predicted to play potential roles in aortic dilation.^[26]^ Carriers of the GG genotype of the rs11643630 polymorphism had significantly smaller aortic diameters than those with GT genotype or TT genotype (shown in Table 4). Three of 4 patients who suffered from a ruptured AD were with TT genotype. No associations were observed between either the rs11644561 polymorphism or rs243865 polymorphism and AD size.

**Table 4:**
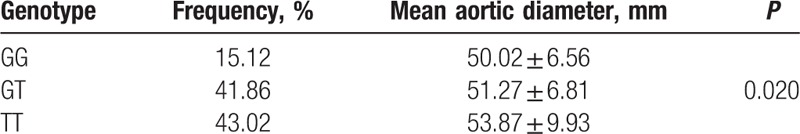
Aortic diameters in type A AD patients according to rs11643630 genotypes.

### 3.4 Identification of haplotypes associated with type A AD

**Figure 1:**
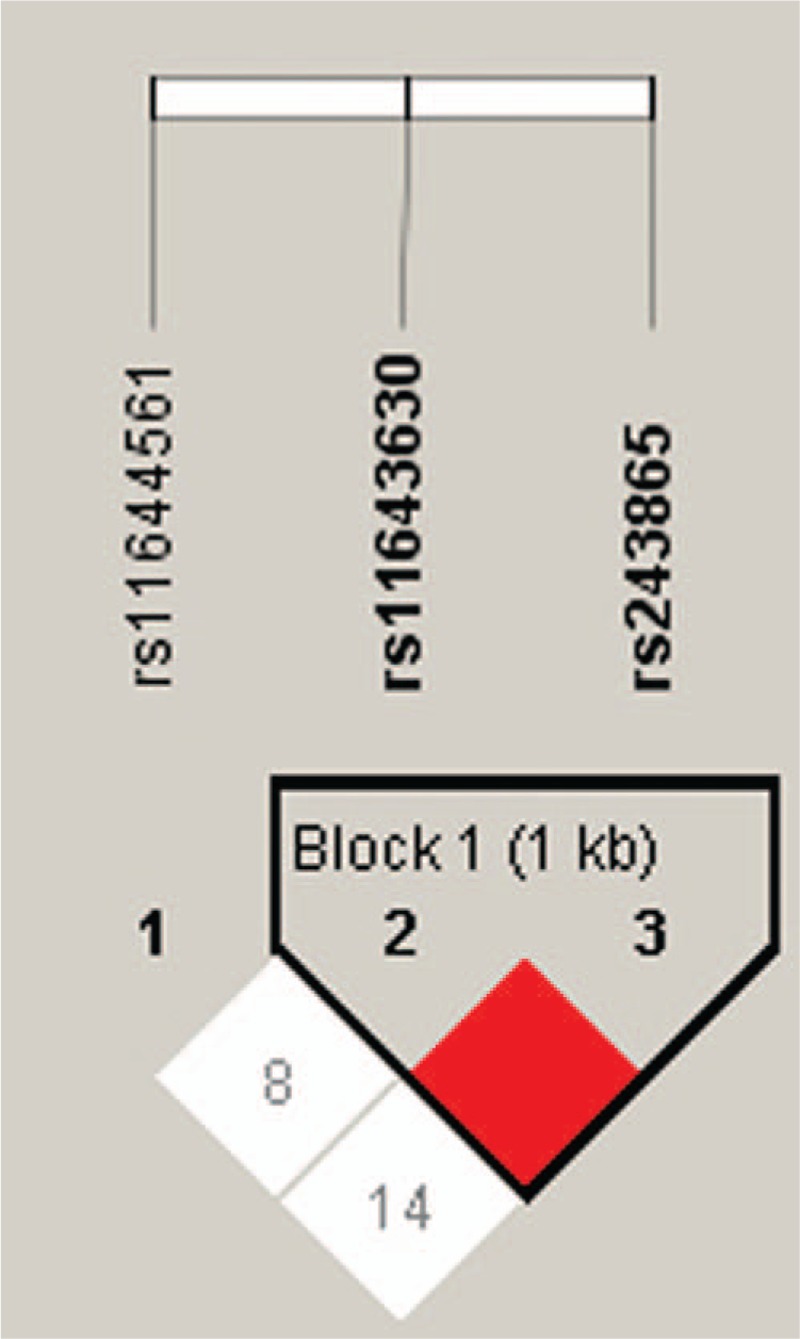
Analysis of linkage disequilibrium patterns between 3 investigated single nucleotide polymorphisms. Red indicates linkage disequilibrium (D = 1, logarithm of odds [LOD] ≥2); white and blue indicate evidence of recombination (D < 1, LOD < 2 for white).

An extended SNP haplotype analysis was conducted to provide some insights into the relation between the SNP patterns and type A AD that is beyond what single point SNP analysis can reveal. Linkage disequilibrium (LD) blocks were constructed among the SNPs based on the default algorithm of Gabriel et al.^[27]^ As shown in Fig. 1, 1 haplotype block was defined. Table 5 showed the haplotype frequency distributions in patients and controls. The haplotypes, TC and GC, showed strong associations with type A AD. Haplotype TC conferred a significant risk effect (*P* = 0.021 and OR = 1.35), whereas the GC haplotype conferred protection of type A AD (*P* = 0.008 and OR = 0.70). Further in multivariate logistic regression analysis, both haplotypes remained significantly associated with type A AD (Table 5). However, only association of haplotype GC with AD remains significant after random permutations (*P* = 0.036).

**Table 5:**
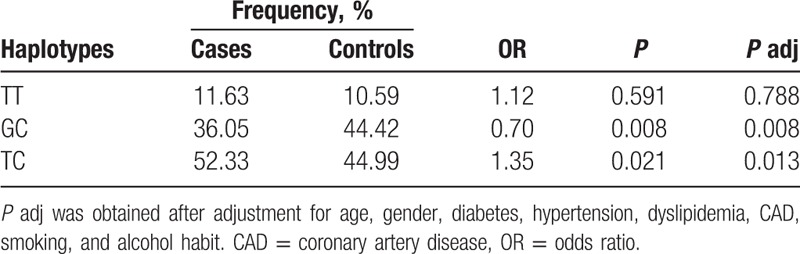
Data on haplotypes consisting of rs11643630 and rs243865.

Concerning the effects of the rs11643630 polymorphism significantly associated with type A AD on aortic diameters, we further identify the association between haplotypes and aortic diameters in type A AD patients. Again, a significant correlation was observed between haplotype GC and AD size (*P* = 0.020).

## 4 Discussion

MMP-2 is a zinc-dependent peptidase that belongs to the gelatinase subfamily of MMPs.^[28]^ Multiple studies in animal models and clinical studies have shown that the actions of MMP-2 contribute to the development of AD.^[29,30]^ In the present study, we provided genetic evidence to suggest that MMP-2 was associated with type A AD. In view of the disease association found in the study of breast cancer,^[17]^ we analyzed 3 SNPs of *MMP-2* gene in a Chinese Han population. We observed an association between the rs11643630 SNP and the occurrence of type A AD. In addition, this genetic locus was strongly associated with aortic diameter in type A AD patients. In addition, 1 haplotype of the *MMP-2* gene showed significant differences in frequencies between cases and controls. Our results suggest that the polymorphism of *MMP-2* gene plays an important role in susceptibility to type A AD in Chinese Han population.

SNP rs11643630 is located approximately 4 and 2.6 kb upstream of the MMP-2 transcription initiation site. This region does not contain any obvious regulatory elements. With current evidence, we still could not determine whether this genetic locus represents novel functional SNP that may affect *MMP-2* gene expression.^[17]^ However, rs11643630 may involve in the disease in a complicated way. LD between this SNP and other causative SNPs should not be ruled out. It is possible that the functional SNP is not rs11643630, but a SNP in linkage disequilibrium with rs11643630.

In previous study, we found that 2 SNPs, rs2241145 and rs9928731, in *MMP-2* gene were associated with thoracic AD, while the effect of rs11643630 was not significant.^[25]^ However, in the present study we found that rs11643630 was associated with type A AD and aortic diameters in patients. As we know, one of commonly used classification schemes for thoracic AD is Stanford scheme. In the Stanford classification, TAD could be divided into type A dissections, which involve the ascending aorta, and type B dissections, which do not, regardless of point of origin.^[31]^ It has been reported that different sites of AD have different embryological origins.^[32]^ The ascending aorta is derived from neural crest, while the descending aorta is paraxial/somitic mesoderm derived.^[32]^ Such a difference in embryological origins predicts the differential MMP activation between ascending and descending aorta.^[32]^ So, both embryological origins and genetic susceptibility could contribute to the expression of MMP-2 and the development of AD. In our previous study, both type A and B AD patients comprised our study population, whereas only type A AD patients were included in the present study. So, the different embryological origins of type A and B AD could explain the discrepancy between these 2 studies.

The main value, from a clinical standpoint, of this SNP is possibly not that of a “genetic screening marker”. Carriers of the GG genotype of the rs11643630 polymorphism had significantly smaller aortic diameters than those with GT or TT genotype. It is known that the risk of AD and rupture rises with increasing aortic diameter.^[33,34]^ The findings in our study suggest that the rs11643630 polymorphism is related to the aortic dilation in type A AD patients. In addition, 3 of 4 patients who suffered from a ruptured AD were with TT genotype. It indicates that genetic factors should be considered when we make treatment protocol for type A AD patients. The patients with certain genotype of the *MMP-2* gene may be prone to suffer from aortic rupture and need emergency surgical operation. In other words, the genomic polymorphisms appeared to be useful for predicting the outcome of type A AD.

If clinical information is available, it should be useful to relate aortic diameter to body mass index (BMI). As we all know, BMI could be calculated using the formula weight (kg)/height (m^2^).^[35]^ However, in clinical practice it is really difficult to measure the TAD patient's weight and height. Because strict bed rest in a quiet room is essential for the management of TAD patients to prevent aortic rupture,^[36]^ we did not record BMI in this study concerning the safety. Even though we still believe that aortic diameter alone could be powerful enough to evaluate the severity of TAD. As described in most clinical guidelines, cardiovascular surgeons just use aortic diameter to predict aortic rupture and to better determine the best timing for surgery.^[31,36]^ For example, the risk of rupture increases sharply with aortic diameters >6 cm at the ascending aorta.^[36]^ Based on these evidence, we believe that the rs11643630 polymorphism identified to be associated with aortic diameters in TAD patients should be a useful predictor of rupture for AD.

*MMP-2* has been considered as a target candidate gene which plays an important role in numerous human diseases.^[37,38]^ As was mentioned in the introduction, association between these 3 MMP-2 SNPs and breast cancer was recently reported.^[17]^ However, we failed to find any significant difference between cases and controls with SNPs rs11644561 and rs243865. There are several underlying mechanisms explaining this discrepancy. First, the key mechanisms of type A AD and breast cancer are not exactly the same. For example, the immune system involves in the development of breast cancer, but does not involve in AD formation.^[39,40]^ Second, because only Chinese women were included in the breast cancer study, the association between the tested SNPs and breast cancer formation may be gender-specific. In our study, both women and men comprised the study population. Third, as a complex disease, environmental factors are involved in the pathogenesis of AD.^[41]^ In addition, different environmental factors between 2 study populations may account for the divergence between these 2 studies.

We should recognize the limitation of this study because of the gender difference between patients and controls. Consistent with others’ observations,^[42,43]^ there were significantly more male type A AD patients than female patients in this study. In order to minimize the influence of gender difference, we utilized multivariate logistic regression analysis to adjust effects of clinical covariates. The association remained significant after adjusting for clinical covariates including gender. Based on the significant findings in logistic regression analysis, we believe that the risk estimation we report is valid.

In conclusion, our case–control study results suggest that polymorphisms in *MMP-2* gene contribute to type A AD susceptibility. In addition, MMP-2 SNPs are associated with AD size. Our findings can provide context for better understanding of the genetic and molecular pathogenesis of type A AD. *MMP-2* gene polymorphisms may be used not only as a prognostic marker in type A AD, but also as a target for the development of new therapeutic approaches.
